# Impact of Chronic Periodontitis on Levels of Glucoregulatory Biomarkers in Gingival Crevicular Fluid of Adults with and without Type 2 Diabetes

**DOI:** 10.1371/journal.pone.0127660

**Published:** 2015-05-20

**Authors:** Hasaan G. Mohamed, Shaza B. Idris, Manal Mustafa, Mutaz F. Ahmed, Anne N. Åstrøm, Kamal Mustafa, Salah O. Ibrahim

**Affiliations:** 1 Department of Clinical Dentistry, Faculty of Medicine and Dentistry, University of Bergen, Bergen, Norway; 2 Department of Oral Rehabilitation, Faculty of Dentistry, University of Khartoum, Khartoum, Sudan; 3 Oral Health Competence Center in Western Norway, Hordaland, Bergen, Norway; 4 Hamad Medical Corporation, Doha, Qatar; University of North Carolina at Chapel Hill, UNITED STATES

## Abstract

The relationship between diabetes and periodontal disease is bidirectional, but information about the effect of chronic periodontitis on the levels of the glucoregulatory biomarkers locally in gingival crevicular fluid (GCF) is limited. The aim of this study was to compare the levels of 10 glucoregulatory biomarkers in GCF, firstly in subjects with type 2 diabetes (T2DM) presenting with and without chronic periodontitis and secondly, in subjects without diabetes, with and without chronic periodontitis. The material comprised a total of 152 subjects, stratified as: 54 with T2DM and chronic periodontitis (G1), 24 with T2DM (G2), 30 with chronic periodontitis (G3) and 44 without T2DM or periodontitis (G4). The levels of the biomarkers were measured using multiplex biometric immunoassays. Periodontal pocket depths were recorded in mm. Subsets G1 and G2 and subsets G3 and G4 were compared independently. Among T2DM subjects, GIP, GLP-1 and glucagon were significantly up-regulated in G1 compared to G2. Moreover, there were no statistical differences between the two groups regarding C-peptide, insulin, ghrelin, leptin and PAI-1. Comparisons among individuals without T2DM revealed significantly lower amounts of C-peptide and ghrelin in G3 than in G4. The number of sites with pocket depth ≥ 4mm correlated negatively with C-peptide (Spearman’s correlation co-efficient: -0.240, P < 0.01) and positively with GIP and visfatin (Spearman’s correlation co-efficient: 0.255 and 0.241, respectively, P < 0.01). The results demonstrate that chronic periodontitis adversely influences the GCF levels of glucoregulatory biomarkers, as it is associated with disturbed levels of biomarkers related to the onset of T2DM and its medical complications.

## Introduction

Diabetes mellitus represents a heterogeneous group of metabolic disorders associated with disturbances of carbohydrate, fat and protein metabolism. There are two major classes of the disease: Type 1 and Type 2. Type 2 diabetes (T2DM) is characterized by elevated blood glucose levels caused by increased production of glucose in the liver and increased peripheral insulin resistance, which might eventually lead to a reduction in insulin secretion [1]. In sub-Saharan Africa, T2DM is becoming increasingly prevalent, presenting a major public health burden in countries with scarce resources [2]. In The Sudan, the prevalence is increasing to epidemic proportions, affecting about 18 percent of the population [3]. Of particular concern is the fact that the onset is insidious and the condition may remain undiagnosed for a long time. In this context, the dental profession has a potentially important role and the condition may first be identified during a dental examination [4].

Periodontal disease is one of the most common diseases affecting humans [5]. Chronic periodontitis is an inflammatory condition characterized by apical migration of junctional epithelium, leading to formation of deep periodontal pockets around the teeth due to destruction of periodontal tissue fibers and the supporting bone [6]. Periodontal pockets may host highly virulent microorganisms and if left untreated; the host inflammatory-immune response progresses leading to tissue destruction [7]. For many years, chronic periodontitis was regarded as a local inflammatory condition, but recent research confirms that the condition has a systemic impact as the total surface area of the ulcerated periodontal pockets may be in the range of 8 to 20 cm^2^, approximately the size of the palm of an adult hand [8]. Recently, more attention is directed towards the systemic effect of chronic periodontitis including its role in the aetiology of T2DM as well as its impact on the metabolic control and medical complications associated with patients baring the disease.

The scientific literature however, shows lack of consensus about the risk of onset of T2DM in subjects with chronic periodontitis. A review by Sima *et al*., [9] cited two longitudinal studies. The first study reported an odds ratio between 1.5 and 2.1 for patients with high periodontal index scores or tooth loss at baseline to develop diabetes [10], while the second one found no association between periodontal disease and the incidence of diabetes [11].

Gingival crevicular fluid (GCF) is a serum transudate found in the gingival sulcus. It contains not only connective tissue degradation products but also inflammatory molecules derived from circulating blood [12]. The levels of biomarkers in the GCF are potentially important as predictors of disease progression [7]. Few studies have investigated the levels of diabetes-related biomarkers in GCF such as ghrelin [13], leptin [14], and visfatin [15]. In the present study, the multiplex biometric immunoassay technique was applied in order to determine the amounts of 10 diabetes-related protein biomarkers known to be involved in the regulation of glucose metabolism, namely: *C-peptide*, *ghrelin*, *gastric inhibitory polypeptide* (GIP), *glucagon-like peptide-1* (GLP-1), *glucagon*, *insulin*, *leptin*, *plasminogen activator inhibitor-1* (PAI-1), *resistin* and *visfatin*.

The aim of the study was to investigate the influence of chronic periodontitis on the levels of glucoregulatory biomarkers in the GCF, by comparing the levels of the above biomarkers in subjects with T2DM, with and without chronic periodontitis, and in subjects without diabetes, with and without chronic periodontitis. We tested the hypothesis that in patients with T2DM, chronic periodontitis interferes with the regulation of glucose metabolism and that this effect is reflected in the relative quantities of the corresponding glucoregulatory biomarkers in the GCF, compared to the levels in subjects with T2DM who do not have chronic periodontitis. The second hypothesis tested was that in subjects without T2DM, chronic periodontitis is associated with increased GCF levels of biomarkers known for their association with the onset of T2DM.

## Materials and Methods

### Subjects and study design

The sample population comprised a total of 152 participants, 78 with T2DM and 74 without T2DM, representing a randomly selected subset from 461 participants recruited for a previous study by Mohamed *et al*. [16]. The T2DM subjects were stratified according to periodontal status, into two groups: 54 with chronic periodontitis (G1) and 24 without periodontitis (G2). Subjects without T2DM were also stratified according to periodontal status, into two groups: 30 with chronic periodontitis (G3) and 44 without periodontitis (G4). The T2DM subjects were recruited from The Jaber Abol’ez Diabetes Center in Khartoum-Sudan. T2DM was diagnosed by specialist physicians at the center according to the criteria of The American Diabetes Association [17]. Briefly, diagnosis of T2DM was based on one of the following: fasting plasma glucose of ≥ 126 mg/dl (≥ 7.0 mmol/L), random plasma glucose of ≥ 200 mg/dl (≥ 11.1 mmol/L), or plasma glucose of ≥ 200 mg/dl (≥ 11.1 mmol/L) after 75 g oral glucose tolerance test. A glycated haemoglobin test (HbA1c) was undertaken at the center’s laboratory, using a commercially available kit (LabonaCheck A1c analyzer). Subjects without T2DM were recruited from the out-patient dental clinic at the Khartoum Dental Teaching Hospital. Recruitment of the study participants and the eligibility criteria for enrolment have been described in detail elsewhere [16]. Briefly, the criteria for enrolment were (i), being diagnosed with T2DM for more than one year and attending a specialized diabetes clinic-for the T2DM patients- (ii), having at least for 10 remaining teeth (iii), no antibiotic, no steroidal and/or non-steroidal anti-inflammatory medication used during the last 3 weeks (iv), not treated with immunosuppressive chemotherapy, no current acute illness, no professional periodontal treatment received during the last 6 months and no ongoing pregnancy or lactation.

The study protocol was approved by the Ministry of Health in the Sudan and by the Norwegian Research Ethics Committee at the University of Bergen (2012/1470/REK Vest). Participation was confirmed by written informed consent and the steps of the clinical examination and sampling procedures were explained to each participant. All participants were provided with oral and written oral hygiene instructions, informed of their dental diagnosis and referred for appropriate dental treatment if needed.

### Clinical examination

Periodontal examination included all teeth except 3^rd^ molars. All participants were examined by a single, calibrated examiner (HGM) using a color-coded periodontal probe (N22, 2-4-6-8-10-12 mm markings), a color-coded Nabors furcation probe (NAB2, 3-6-9-12 mm markings), curette, mirror, probe, tweezers and cotton rolls. Dental plaque was recorded according to the Silness and Loe Index [18]. Periodontal pocket depths (PD) were measured from the gingival margin to the base of the periodontal pocket (mm) at four sites on each tooth (mesial, distal, buccal and lingual). Subjects with at least two sites with PD of ≥ 4 mm (not on the same tooth) with bleeding on probing were diagnosed as having chronic periodontitis [19,20]. It was reported that both periodontal pocket depth and bleeding on probing reflect the current disease status and are strongly related to the local inflammatory activity [21,22].

### GCF sampling

Before the clinical examination, GCF samples were collected on the same day, using paper strips (PERIOPAPER Gingival Fluid Collection Strips, Oraflow Inc, New York, USA). Four samples, representing the four quadrants, were collected from each participant. Samples were collected from the mesiobuccal site of the sulcus/pocket. The 1^st^ molar or, if missing, the 2^nd^ molar, 2^nd^ premolar or 1^st^ premolar was sampled respectively. If the target teeth were missing in one quadrant, then no GCF sample was collected from that quadrant. After removing the supra-gingival biofilm with sterile cotton pellets, the sites were dried and isolated with cotton rolls to avoid saliva contamination. To collect the GCF, the paper strips were inserted 2 mm into the sulcus/pocket and left in place for 30 seconds. Strips that were visually assessed as contaminated with blood or saliva were discarded. The four strips were immediately pooled in one tube, labeled and stored in liquid nitrogen until analysed.

### Protein extraction and quantification

For protein extraction, Tween buffer was added to each GCF sample (4 strips). The tubes were kept in a shaker for 30 minutes and then centrifuged for 10 minutes at 4°C and 1400 rpm. The extracted protein was quantified using a commercially available kit (Pierce BCA Protein Assay Kit, Thermoscientific, Rockford, USA) following the manufacturer’s instructions. Absorbance was measured at 560 nm on a plate reader (FLUOstar OPTIMA- BMG Labtech, Germany) and the total protein per sample (4 strips) was calculated in micrograms (μg).

### Multiplex biomarkers assay

Multiplex biometric immunoassay containing fluorescent dyed magnetic beads conjugated with monoclonal antibodies specific for the targeted 10 protein biomarkers was used according to the manufacturer's instructions (Bio-Plex Human Cytokine Assay; Bio-Rad Inc., Hercules, CA, USA) [23]. Following protein extraction and purification, GCF samples were diluted to a ratio of 1:4 and incubated with the magnetic beads. After a series of washes with Bio-Plex Pro wash station, biotinylated detection antibody was added to create a sandwich complex. Thereafter, Streptavidin-Phycoerythrin conjugate was added as a fluorescent indicator. A range of (352290–0.97) pg/ml recombinant cytokines was used to establish the standard curves. Biomarkers quantities were determined using a multiplex array reader powered by Luminex-200 System. The amounts were calculated using Bio-Plex Manager software and reported as picograms per 30 seconds (pg/30 s) [24].

### Statistical analysis

Comparisons were made between the T2DM groups (G1 and G2) and between the groups without T2DM (G3 and G4) independently. Distributions of the continuous variables among each group were assessed using Kolmogorov-Smirnov test. Independent sample-T test, Mann-Whitney U test, chi-square test and Fisher’s Exact test were used to investigate the differences in the distribution of demographic data between the study groups. Quantities of the detected biomarkers were compared between the study groups using Mann-Whitney U test. Logistic regression analysis adjusting for age, gender, dental plaque and total protein was applied to test the relationship between periodontal status and the quantities of biomarkers. Spearman’s correlation coefficients were calculated to assess the correlation between the number of sites with PD ≥ 4 mm and the amount of each biomarker. Stata 13 (Stata Corp. 2013, Stata Statistical Software: Release 13. College Station, TX: StataCorp LP) was used for statistical analysis. P values less than 0.05 were considered statistically significant.

## Results

As presented in Table 1, the mean ages of the T2DM patients in G1 and G2 were 54.76 ± 1.38 and 50.79 ± 2.09 (P >0.05), respectively. The age ranges were 24–70 and 33–70 years, respectively. There were no significant intergroup differences between G1 and G2 in the demographic and clinical indicators. Oral hypoglycemic drugs were the most common drugs for treatment of diabetes in G1 (66.7%) and insulin in G2 (50.0%). In subjects without T2DM, the mean ages of G3 and G4 were 55.37 ± 1.77 and 47.15 ± 1.56 (P <0.01), respectively. The age ranges were 38–70 and 24–65 years, respectively. Plaque index was the only clinical indicator that differed significantly between G3 and G4. Among those with chronic periodontitis, the PD ranged from 4 to 10 mm in G1, and from 4 to 6 mm in G3, while participants without chronic periodontitis (G2 and G4) had a normal gingival sulcus ranged from 1–3 mm.

**Table 1:** Demographic characteristics and clinical indicators of the study groups.

	Type 2 diabetes			No diabetes		
Variable	Chronic periodontitis (G1), n = 54	No periodontitis (G2), n = 24	P value	Chronic periodontitis (G3), n = 30	No periodontitis (G4), n = 44	P value
**Age, mean (SE)**	54.76 (1.38)	50.79 (2.09)	0.12^a^	55.37 (1.77)	47.15 (1.56)	<0.01^a^
**Gender, % (n)**		)		)		
Male	42.60 (23)	29.20 (7)	0.38^b^	60.00 (18)	34.10 (15)	0.05^b^
Female	57.40 (31)	70.80 (14)		40.00 (12)	65.90 (29)	
**Education, % (n)**						
Literate	70.37 (38)	79.17(19)	0.42^b^	66.67 (20)	77.27 (34)	0.31^b^
Illiterate	29.63 (16)	20.83 (5)		33.33 (10)	22.73 (10)	
**Employment, % (n)**						
				)	)	
Employed	37.04 (20)	29.17 (7)	0.50^b^	56.67 (17)	36.36 (16)	0.09^b^
Unemployed	62.96 (34)	70.83 (17)		43.33 (13)	63.64 (28)	
**Smoking, % (n)**		)				
Yes	13.00 (7)	12.50 (3)	1.00^c^	26.70 (8)	13.60 (6)	0.27^b^
No	87.00 (47)	87.50 (21)		73.30 (22)	86.40 (38)	
**Plaque index, mean (SE)**	1.66 (0.05)	1.49 (0.05)	0.05^a^	1.49 (0.06)	1.28 (0.06)	0.03^a^
**Pocket depth, % (n)**						
4–5 mm	59.30 (32)	0.00 (0)	——-	70.00 (21)	0.00 (0)	——-
≥ 6 mm	40.70 (22)	0.00 (0)		30.00 (9)	0.00 (0)	
**Total protein -μg, mean (SE)**	82.78 (7.53)	77.53 (9.06)	0.92^d^	84.23 (15.25)	86.50 (9.51)	0.25^d^
**Duration of T2DM, mean (SE)**	8.44 (0.83)	9.67 (1.70)	0.61^d^	——-	——-	——-
**T2DM medication used, % (n)**		)		——-	——-	——-
Dilatory control	0.00 (0)	12.50 (3)	<0.01^c^			
Oral-hypoglycemic	66.70 (36)	33.30 (8)				
Insulin	29.60 (16)	50.00 (12)				
Both	3.70 (2)	4.20 (1)				
**HbA1c %, mean (SE)**	9.17 (0.24)	9.25 (0.49)	0.89^a^	——-	——-	——-

^**a**^ Independent sample-T test.

^**b**^ Chi-square test.

^**c**^ Fisher’s Exact test.

^**d**^ Mann-Whitney U test.

**n**: sample size, **SE**: standard error of the mean, **T2DM**: type 2 diabetes, **HbA1c**: glycated haemoglobin.

Of the 10 glucoregulatory biomarkers investigated in this study, resistin was excluded from the analysis, as it was not detected in 60% of the GCF samples. The mean quantities of the detected diabetes-related biomarkers among the study groups are presented in Table 2.

**Table 2:** Amounts of the detected glucoregulatory biomarkers (pg/30 s).

	Type 2 diabetes			No diabetes		
Biomarker, mean (SE)	Chronic periodontitis (G1),	No periodontitis (G2),	P value^a^	Chronic periodontitis (G3),	No periodontitis (G4),	P value^a^
	n = 54	n = 24		n = 30	n = 44	
C-peptide	26.22 (0.87)	25.27 (1.55)	0.82	35.15 (1.91)	42.98 (2.52)	0.10
Insulin	24.36 (1.38)	20.02 (1.94)	0.12	25.59 (1.51)	30.92 (2.28)	0.17
GIP	164.21 (3.35)	144.64 (4.54)	<0.01	141.45 (3.76)	151.27 (3.14)	0.07
GLP-1	210.99 (5.27)	183.40 (7.65)	<0.01	188.63 (7.42)	203.02 (6.52)	0.30
Glucagon	261.77 (8.06)	223.82 (10.72)	0.01	242.91 (9.40)	250.17 (7.29)	0.60
Ghrelin	303.63 (15.82)	239.00 (19.34)	0.04	303.20 (19.00)	374.22 (21.91)	0.03
Leptin	85.85 (5.52)	64.78 (5.49)	0.05	84.46 (6.36)	99.57 (6.66)	0.14
PAI-1	1027.19 (155.80)	901.53 (260.49)	0.32	512.54 (86.75)	619.04 (110.98)	0.18
Visfatin	6411.37 (471.25)	5976.73 (1201.05)	0.13	7013.91 (978.87)	5194.03 (414.78)	0.26

^**a**^ Mann-Whitney U test.

**n**: sample size, **SE**: standard error of the mean, **GIP**: gastric inhibitory polypeptide, **GLP-1**: glucagon-like peptide-1, **PAI-1**: plasminogen activator inhibitor-1.

After adjustment for potential confounders, GIP, GLP-1 and glucagon were significantly higher in G1 than in G2. Moreover, C-peptide, insulin, ghrelin, leptin and PAI-1 were insignificantly higher in G1 than in G2 (Table 3). In contrast, in subjects without T2DM, the levels of C-peptide and ghrelin were significantly higher in G4 than in G3. All the other biomarkers under investigation were insignificantly higher in G4 than in G3, except visfatin which was insignificantly higher in G3 (Table 3).

**Table 3:** Logistic regression analysis with periodontal status as dependent variable (yes/no).

	Type 2 diabetes			No diabetes		
Biomarker	Coefficient (SE)	OR (95% CI)	P value^a^	Coefficient (SE)	OR (95% CI)	P value^a^
C-peptide	0.008 (0.041)	1.008 (0.931–1.092)	0.85	-0.053 (0.023)	0.948 (0.907–0.992)	0.02
Insulin	0.060 (0.033)	1.062 (0.996–1.133)	0.07	-0.049 (0.027)	0.952 (0.902–1.005)	0.07
GIP	0.035 (0.014)	1.035 (1.008–1.064)	0.01	-0.028 (0.016)	0.972 (0.942–1.003)	0.08
GLP-1	0.022 (0.010)	1.022 (1.003–1.042)	0.02	-0.009 (0.007)	0.991 (0.977–1.006)	0.12
Glucagon	0.013 (0.006)	1.013 (1.001–1.025)	0.03	-0.003 (0.006)	0.997 (0.986–1.008)	0.60
Ghrelin	0.005 (0.003)	1.005 (0.999–1.010)	0.11	-0.006 (0.003)	0.994 (0.989–0.999)	0.02
Leptin	0.016 (0.010)	1.016 (0.997–1.036)	0.09	-0.015 (0.008)	0.985 (0.970–1.001)	0.06
PAI-1	0.006 (0.024)	1.006 (0.959–1.054)	0.82	-0.079 (0.058)	0.924 (0.825–1.035)	0.17
Visfatin	-0.001 (0.006)	0.999 (0.986–1.011)	0.85	0.009 (0.008)	1.009 (0.993–1.025)	0.27

^**a**^ Adjusted for age, gender, plaque index and total GCF protein.

**OR**: adjusted odds ratio, **CI**: confidence interval, **SE**: standard error, **GIP**: gastric inhibitory polypeptide, **GLP-1**: glucagon-like peptide-1, **PAI-1**: plasminogen activator inhibitor-1.

As the number of diseased sites increased (PD ≥ 4 mm), the amount of C-peptide decreased significantly (Spearman’s correlation co-efficient: -0.240). In contrast, the amounts of both GIP and visfatin increased significantly as the number of diseased sites increased (Spearman’s correlation co-efficient: 0.255 and 0.241, respectively). We also observed a weakly positive correlation between the number of sites with PD ≥ 4 mm and the amounts of both GLP-1 and glucagon (Spearman’s correlation co-efficient: 0.131 and 0.153, respectively) (Table 4).

**Table 4:** Correlations between number of sites with PD ≥ 4mm and the amounts of the detected biomarkers.

Biomarker	Correlation coefficient^a^	P value
C-peptide	-0.240	<0.01
Insulin	-0.005	0.95
GIP	0.255	<0.01
GLP-1	0.131	0.11
Glucagon	0.153	0.06
Ghrelin	-0.017	0.84
Leptin	0.030	0.72
PAI-1	0.081	0.32
Visfatin	0.241	<0.01

^**a**^ Spearman’s correlation.

**GIP**: gastric inhibitory polypeptide, **GLP-1**: glucagon-like peptide-1, **PAI-1**: plasminogen activator inhibitor-1.

## Discussion

There is ample evidence from the scientific literature that the relationship between diabetes and chronic periodontitis is bidirectional [25,26]. However, the focus of this report was to investigate the impact of chronic periodontitis on levels of glucoregulatory biomarkers in the GCF. In the present study, chronic periodontitis adversely influenced the levels of glucoregulatory biomarkers in the GCF, indicating that it might be associated with the onset of T2DM and the risk of diabetes related medical complications in subjects with the disease. Multiplex technology has been applied in a few studies, to investigate diabetes-related biomarkers in patients with systemic conditions such as obesity [27] and gestational diabetes [28]. In the present study, this high-throughput technology provided a highly accurate analysis of biological samples, given that it requires less sample volume and is able to detect different proteins within a broad range of concentrations [29]. When tested on the same analytics, multiplex assays are reported to compare favorably with ELISA with respect to sensitivity, accuracy, and reproducibility [30].

C-peptide is a polypeptide secreted from the secretory granules of β-cells during the conversion of pro-insulin to insulin [31]. C-peptide and insulin are released into the circulation in comparable amounts, but C-peptide is a more reliable indicator of β-cell activity, as it has a longer half-life and is not affected by insulin-specific antibodies [32]. To date, there are no published data on the relationship between periodontal disease and C-peptide levels in adults with T2DM. However, Merchant *et al*., [33] investigated such an association in a sample comprising young subjects with both type 1 and 2 diabetes and concluded that periodontal disease was associated with lower fasting C-peptide. We observed the same trend among subjects without T2DM, as the level of C-peptide was lower among subjects with periodontitis (G3) than in those without periodontitis (G4). Moreover, the amount of C-peptide was negatively correlated with the number of diseased sites with PD ≥ 4 mm, suggesting that chronic periodontitis might be associated with disturbed β-cells activity. In the present study, comparisons of insulin among the different study groups showed similar trends, but there was no correlation between insulin and the severity of periodontal disease.

GLP-1 and GIP are insulin-stimulating hormones secreted from the gastrointestinal system after food intake [34]. Patients with T2DM do not generally exhibit reduced GLP-1 secretion [35]. In contrast, results from studies comparing GIP levels in T2DM patients and systemically healthy controls were inconclusive [34]. In the present study, both GIP and GLP-1 were found to be significantly higher in G1 than in G2. This observation may be attributable to the fact that 50% of patients in G2 were under insulin therapy, compared to 29.6% in G1. Thus, exogenous insulin might have down-regulated the insulin-stimulating hormones by negative feed-back. Moreover, the levels of GIP and GLP-1 were lower in G3 than in G4. Despite the statistical insignificance, these findings support the hypothesis that chronic periodontitis is associated with disturbed GCF levels of hormones responsible for regulating the blood glucose levels among individuals without T2DM.

Glucagon is a hormone secreted by α-cells in response to food intake and hypoglycemia [36]. It regulates blood glucose levels and counteracts the effect of insulin by increasing blood glucose levels. In T2DM, the feed-back mechanism which controls glucagon secretion in response to blood glucose level seems to malfunction [37]. Thus, glucagon remains inappropriately elevated in hyperglycemia at comparable levels of blood glucose. Our results indicated no statistical difference in glucagon between G3 and G4. In subjects with T2DM however, the amount of glucagon was higher in G1 than in G2. This finding suggests that in T2DM subjects, chronic periodontitis may exert a systemic effect by its association with increased glucagon secretion.

Ghrelin is a peptide hormone secreted from the oral epithelium and fibroblasts. It has a major role in regulating neutrophil-mediated innate immune response [13]. Low ghrelin concentration is associated with T2DM [38]. The amount of ghrelin in G3 was significantly lower than in G4. Yilmaz *et al*., [39] reported inconclusive results in a study investigating the level of plasma ghrelin in systemically healthy subjects with and without periodontitis. In the present study, the opposite trend was observed in the T2DM groups, as those with chronic periodontitis had higher levels of ghrelin than those without periodontitis. One explanation is that there might be a synergistic systemic effect of both T2DM and chronic periodontitis, as high ghrelin levels have been reported in other chronic inflammatory diseases such as Crohn’s disease and inflammatory bowel disease [40].

Leptin is a hormone secreted by adipose tissues. It exerts an anti-diabetic effect by reducing insulin resistance [41]. The leptin detected in GCF probably diffuses from the microvasculature to the gingival tissues, as there are no adipocytes within the gingival tissue [14]. Tatti *et al*., [42] have reported higher leptin concentration in systemically healthy individuals than in those with T2DM. In contrast, earlier investigations comparing Sudanese adults with and without diabetes have reported elevated plasma leptin levels in subjects with diabetes [43]. The present findings with respect to subjects without T2DM are consistent with earlier reports of higher levels of leptin in periodontally healthy subjects than in those with periodontal disease [14,44]. This has been attributed to the fact that during inflammation, the amount of leptin decreases because the increased vascular permeability allows leptin to escape from the gingival tissue [44]. In this context, it is noteworthy that detection of high local leptin levels in T2DM patients with periodontal disease is regarded as one of the indicators of cardiovascular complications [45]. PAI-1 is a serine protease inhibitor secreted by endothelial cells, fibroblasts, liver and adipose tissue [46]. Increased PAI-1 concentration is reported to be associated with venous thrombosis, pulmonary embolism and the aetiology of T2DM [47,48]. In the present study, the results of PAI-1 were insignificant. Visfatin is an adipokine secreted mainly by visceral adipose tissue [49]. It is also secreted by neutrophils in response to pathogens and acts as a pro-inflammatory cytokine which stimulates monocytes to produce inflammatory mediators [50]. In the present study, there was a significant positive correlation between PD and visfatin, a finding which is in concordance with a previous report by Pradeep *et al* [15].

It was not possible to measure the GCF volume. Instead, we used total GCF protein as a surrogate measure of the GCF volume in the multivariate analysis as an attempt to control for the potential effect of variability of GCF volume on our results [51]. Data on the study participant’s height and weight were unavailable. Thus, we were not able to calculate the body mass index (BMI) which is a proxy variable reflecting the amount of adipose tissue for each participant. This might affect our findings, as some of the biomarkers studied are secreted by adipocytes such as leptin, PAI-1 and visfatin.

Within the limitations of the study, our data imply that chronic periodontitis is associated with disturbance of the local expressions of biomarkers related to the onset of T2DM and its medical complications in GCF. Large-scale longitudinal studies are required to confirm the association between chronic periodontitis and the onset of T2DM and its medical complications.

## References

[1] Mealey BL, Ocampo GL. Diabetes mellitus and periodontal disease. Periodontol 2000. 2007;44: 127–153. 17474930 10.1111/j.1600-0757.2006.00193.x

[2] Hall V, Thomsen RW, Henriksen O, Lohse N. Diabetes in Sub Saharan Africa 1999–2011: epidemiology and public health implications. A systematic review. BMC public health. 2011;11:564 10.1186/1471-2458-11-564 21756350 PMC3156766

[3] IDF Diabetes Atlas update poster, 6th edn Brussels, Belgium International Diabetes Federation 2014.

[4] Lamster IB, Lalla E, Borgnakke WS, Taylor GW. The relationship between oral health and diabetes mellitus. J Am Dent Assoc 2008 10;139(10 Suppl): 19S–24S. 10.14219/jada.archive.2008.0363 18809650

[5] Pihlstrom BL, Michalowicz BS, Johnson NW. Periodontal diseases. Lancet. 2005;366(9499): 1809–1820. 16298220 10.1016/S0140-6736(05)67728-8

[6] Armitage GC. Periodontal diagnoses and classification of periodontal diseases. Periodontol 2000. 2004;34:9–21. 14717852 10.1046/j.0906-6713.2002.003421.x

[7] Hanes PJ, Krishna R. Characteristics of inflammation common to both diabetes and periodontitis: are predictive diagnosis and targeted preventive measures possible? The EPMA journal. 2010 3;1(1): 101–116. 10.1007/s13167-010-0016-3 23199045 PMC3405308

[8] Hujoel PP, White BA, Garcia RI, Listgarten MA. The dentogingival epithelial surface area revisited. J Periodontal Res. 2001 2;36(1):48–55. 11246704 10.1034/j.1600-0765.2001.00011.x

[9] Sima C, Glogauer M. Diabetes mellitus and periodontal diseases. Current diabetes reports. 2013 6;13(3): 445–452. 10.1007/s11892-013-0367-y 23430581

[10] Demmer RT, Jacobs DR, Desvarieux M. Periodontal disease and incident type 2 diabetes results from the first national health and nutrition examination survey and its epidemiologic follow-up study. Diabetes care. 2008;31(7): 1373–1379. 10.2337/dc08-0026 18390797 PMC2453650

[11] Ide R, Hoshuyama T, Wilson D, Takahashi K, Higashi T. Periodontal disease and incident diabetes a seven-year study. Journal of dental research. 2011;90(1): 41–46. 10.1177/0022034510381902 21041549

[12] Lamster IB, Ahlo JK. Analysis of gingival crevicular fluid as applied to the diagnosis of oral and systemic diseases. Annals of the New York Academy of Sciences. 2007 3;1098: 216–229. 17435131 10.1196/annals.1384.027

[13] Ohta K, Laborde NJ, Kajiya M, Shin J, Zhu T, Thondukolam AK, et al Expression and possible immune-regulatory function of ghrelin in oral epithelium. Journal of dental research. 2011 11;90(11): 1286–1292. 10.1177/0022034511420431 21865591 PMC3188459

[14] Karthikeyan BV, Pradeep AR. Leptin levels in gingival crevicular fluid in periodontal health and disease. J Periodontal Res. 2007 8;42(4): 300–304. 17559625 10.1111/j.1600-0765.2006.00948.x

[15] Pradeep AR, Raghavendra NM, Prasad MV, Kathariya R, Patel SP, Sharma A. Gingival crevicular fluid and serum visfatin concentration: their relationship in periodontal health and disease. J Periodontol. 2011 9;82(9): 1314–1319. 10.1902/jop.2011.100690 21309715

[16] Mohamed HG, Idris SB, Ahmed MF, Bøe OE, Mustafa K, Ibrahim SO, et al Association between Oral Health Status and Type 2 Diabetes Mellitus among Sudanese Adults: A Matched Case-Control Study. PloS ONE. 2013;8(12): e82158 10.1371/journal.pone.0082158 24349205 PMC3859584

[17] ADA. Diagnosis and classification of diabetes mellitus. Diabetes Care. 2008 1;31 Suppl 1: S55–60. 10.2337/dc08-S055 18165338

[18] Silness J, Loe H. Periodontal disease in pregnancy. II. Correlation between oral hygiene and periodontal condition. Acta odontologica Scandinavica. 1964 2;22: 121–135. 14158464 10.3109/00016356408993968

[19] Katagiri S, Nagasawa T, Kobayashi H, Takamatsu H, Bharti P, Izumiyama H, et al Improvement of glycemic control after periodontal treatment by resolving gingival inflammation in type 2 diabetic patients with periodontal disease. Journal of diabetes investigation. 2012 8 20;3(4): 402–409. 10.1111/j.2040-1124.2012.00209.x 24843597 PMC4019262

[20] Eke PI, Page RC, Wei L, Thornton-Evans G, Genco RJ. Update of the case definitions for population-based surveillance of periodontitis. J Periodontol. 2012 12;83(12): 1449–1454. 10.1902/jop.2012.110664 22420873 PMC6005373

[21] Zhong Y, Slade GD, Beck JD, Offenbacher S. Gingival crevicular fluid interleukin-1beta, prostaglandin E2 and periodontal status in a community population. Journal of clinical periodontology. 2007 4;34(4): 285–293. 17378884 10.1111/j.1600-051X.2007.01057.x

[22] Lopez R, Dahlen G, Baelum V. Subgingival microbial consortia and the clinical features of periodontitis in adolescents. European journal of oral sciences. 2011 12;119(6): 455–462. 10.1111/j.1600-0722.2011.00875.x 22112031

[23] Houser B. Bio-Rad's Bio-Plex(R) suspension array system, xMAP technology overview. Archives of physiology and biochemistry. 2012 10;118(4): 192–196. 10.3109/13813455.2012.705301 22852821 PMC3469222

[24] Thunell DH, Tymkiw KD, Johnson GK, Joly S, Burnell KK, Cavanaugh JE, et al A multiplex immunoassay demonstrates reductions in gingival crevicular fluid cytokines following initial periodontal therapy. J Periodontal Res. 2010 2;45(1): 148–152. 10.1111/j.1600-0765.2009.01204.x 19602112 PMC5405696

[25] Taylor GW. Bidirectional interrelationships between diabetes and periodontal diseases: an epidemiologic perspective. Ann Periodontol. 2001 12;6(1):99–112. 11887478 10.1902/annals.2001.6.1.99

[26] Lalla E, Papapanou PN. Diabetes mellitus and periodontitis: a tale of two common interrelated diseases. Nature reviews Endocrinology. 2011 12;7(12):738–748. 10.1038/nrendo.2011.106 21709707

[27] Liu MY, Xydakis AM, Hoogeveen RC, Jones PH, Smith EO, Nelson KW, et al Multiplexed analysis of biomarkers related to obesity and the metabolic syndrome in human plasma, using the Luminex-100 system. Clinical chemistry. 2005 7;51(7): 1102–1109. 15976097 10.1373/clinchem.2004.047084

[28] Georgiou HM, Lappas M, Georgiou GM, Marita A, Bryant VJ, Hiscock R, et al Screening for biomarkers predictive of gestational diabetes mellitus. Acta diabetologica. 2008 9;45(3): 157–165. 10.1007/s00592-008-0037-8 18496643

[29] Leng SX, McElhaney JE, Walston JD, Xie D, Fedarko NS, Kuchel GA. ELISA and multiplex technologies for cytokine measurement in inflammation and aging research. The journals of gerontology Series A, Biological sciences and medical sciences. 2008 8;63(8): 879–884. 18772478 10.1093/gerona/63.8.879 PMC2562869

[30] Almaghlouth AA, Cionca N, Cancela JA, Décaillet F, Courvoisier DS, Giannopoulou C, et al Effect of periodontal treatment on peak serum levels of inflammatory markers. Clinical oral investigations. 2014: 1–9. 10.1007/s00784-014-1333-z 24452825

[31] Hoekstra JB, van Rijn HJ, Erkelens DW, Thijssen JH. C-peptide. Diabetes Care. 1982 Jul-Aug;5(4): 438–446. 6759081 10.2337/diacare.5.4.438

[32] Chowta MN, Adhikari PM, Chowta NK, Shenoy AK, D'Souza S. Serum C peptide level and renal function in diabetes mellitus. Indian journal of nephrology. 2010 1;20(1): 25–28. 10.4103/0971-4065.62093 20535267 PMC2878407

[33] Merchant AT, Jethwani M, Choi YH, Morrato EH, Liese AD, Mayer-Davis E. Associations between periodontal disease and selected risk factors of early complications among youth with type 1 and type 2 diabetes: a pilot study. Pediatric diabetes. 2011 9;12(6): 529–535. 10.1111/j.1399-5448.2010.00736.x 21392193

[34] Alssema M, Rijkelijkhuizen JM, Holst JJ, Teerlink T, Scheffer PG, Eekhoff EM, et al Preserved GLP-1 and exaggerated GIP secretion in type 2 diabetes and relationships with triglycerides and ALT. European journal of endocrinology / European Federation of Endocrine Societies. 2013 10;169(4): 421–430. 10.1530/EJE-13-0487 23864340

[35] Calanna S, Christensen M, Holst JJ, Laferrere B, Gluud LL, Vilsboll T, et al Secretion of glucagon-like peptide-1 in patients with type 2 diabetes mellitus: systematic review and meta-analyses of clinical studies. Diabetologia. 2013 5;56(5): 965–972. 10.1007/s00125-013-2841-0 23377698 PMC3687347

[36] Gromada J, Franklin I, Wollheim CB. Alpha-cells of the endocrine pancreas: 35 years of research but the enigma remains. Endocrine reviews. 2007 2;28(1): 84–116. 17261637 10.1210/er.2006-0007

[37] D'Alessio D. The role of dysregulated glucagon secretion in type 2 diabetes. Diabetes, obesity & metabolism. 2011 10;13 Suppl 1: 126–132. 10.1111/j.1463-1326.2011.01449.x 21824266

[38] Pulkkinen L, Ukkola O, Kolehmainen M, Uusitupa M. Ghrelin in diabetes and metabolic syndrome. International journal of peptides. 2010. 10.1155/2010/248948 PMC2911592 20700400

[39] Yilmaz G, Kirzioglu FY, Doguc DK, Kocak H, Orhan H. Ghrelin levels in chronic periodontitis patients. Odontology / the Society of the Nippon Dental University. 2014 1;102(1): 59–67. 10.1007/s10266-012-0100-3 23292319

[40] Karmiris K, Koutroubakis IE, Kouroumalis EA. Leptin, adiponectin, resistin, and ghrelin--implications for inflammatory bowel disease. Molecular nutrition & food research. 2008 8;52(8): 855–866. 18383234 10.1002/mnfr.200700050

[41] Coppari R, Bjorbaek C. Leptin revisited: its mechanism of action and potential for treating diabetes. Nature reviews Drug discovery. 2012 9;11(9): 692–708. 10.1038/nrd3757 22935803 PMC4019022

[42] Tatti P, Masselli L, Buonanno A, Di Mauro P, Strollo F. Leptin levels in diabetic and nondiabetic subjects. Endocrine. 2001 8;15(3): 305–308. 11762704 10.1385/ENDO:15:3:305

[43] Ahmed AM. Evaluation of plasma leptin levels in Sudanese diabetic patients. Egypt Acad J Biolog Sci 2012;4(1): 107–112.

[44] Johnson RB, Serio FG. Leptin within healthy and diseased human gingiva. J Periodontol. 2001 9;72(9): 1254–1257. 11577959 10.1902/jop.2000.72.9.1254

[45] Kang SM, Kwon HM, Hong BK, Kim D, Kim IJ, Choi EY, et al Expression of leptin receptor (Ob-R) in human atherosclerotic lesions: potential role in intimal neovascularization. Yonsei medical journal. 2000 2;41(1): 68–75. 10731922 10.3349/ymj.2000.41.1.68

[46] Lyon CJ, Hsueh WA. Effect of plasminogen activator inhibitor-1 in diabetes mellitus and cardiovascular disease. The American journal of medicine. 2003 12 8;115 Suppl 8A:62S–68S. 14678868 10.1016/j.amjmed.2003.08.014

[47] Schneider DJ, Sobel BE. PAI-1 and diabetes: a journey from the bench to the bedside. Diabetes Care. 2012 10;35(10): 1961–1967. 22996180 10.2337/dc12-0638 PMC3447837

[48] Vague P, Juhan-Vague I, Aillaud MF, Badier C, Viard R, Alessi MC, et al Correlation between blood fibrinolytic activity, plasminogen activator inhibitor level, plasma insulin level, and relative body weight in normal and obese subjects. Metabolism: clinical and experimental. 1986 3;35(3): 250–253. 3081778 10.1016/0026-0495(86)90209-x

[49] Fukuhara A, Matsuda M, Nishizawa M, Segawa K, Tanaka M, Kishimoto K, et al Visfatin: a protein secreted by visceral fat that mimics the effects of insulin. Science (New York, NY). 2005 1 21;307(5708): 426–430. 15604363 10.1126/science.1097243

[50] Moschen AR, Kaser A, Enrich B, Mosheimer B, Theurl M, Niederegger H, et al Visfatin, an adipocytokine with proinflammatory and immunomodulating properties. Journal of immunology. 2007 2 1;178(3): 1748–1758. 17237424 10.4049/jimmunol.178.3.1748

[51] Petropoulos G, McKay IJ, Hughes FJ. The association between neutrophil numbers and interleukin-1alpha concentrations in gingival crevicular fluid of smokers and non-smokers with periodontal disease. Journal of clinical periodontology. 2004 5;31(5): 390–395. 15086622 10.1111/j.1600-051x.2004.00489.x

